# Western Diet-Induced Obesity Modulates the Mammary Fat Pad Microenvironment

**DOI:** 10.3390/cells15121050

**Published:** 2026-06-08

**Authors:** Md Manirujjaman, Maria D. Sanchez-Pino, Jasjeet Singh, Farzeen Nafees, Mrityunjoy Biswas, Ramesh Thylur Puttalingaiah, Soroor Heidari, Dorota Wyczechowska, Jone Garai, Diana C. Polania-Villanueva, Qingzhao Yu, Luis Del Valle, Lucio Miele, Samarpan Majumder, Jovanny Zabaleta, Fokhrul Hossain

**Affiliations:** 1 Department of Genetics, Louisiana State University Health Sciences Center-New Orleans (LSUHSC-NO), New Orleans, LA 70112, USA; mmanir@lsuhsc.edu (M.M.); mbisw2@lsuhsc.edu (M.B.); lmiele@lsuhsc.edu (L.M.); smaju1@lsuhsc.edu (S.M.); 2 Department of Interdisciplinary Oncology, Louisiana State University Health Sciences Center-New Orleans (LSUHSC-NO), New Orleans, LA 70112, USA; msanc2@lsuhsc.edu (M.D.S.-P.); rthylu@lsuhsc.edu (R.T.P.); dwycze@lsuhsc.edu (D.W.); jgarai@lsuhsc.edu (J.G.); dpolan@lsuhsc.edu (D.C.P.-V.); ldelva@lsuhsc.edu (L.D.V.); jzabal@lsuhsc.edu (J.Z.); 3 School of Medicine, Louisiana State University Health Sciences Center-New Orleans (LSUHSC-NO), New Orleans, LA 70112, USA; jsin14@lsuhsc.edu; 4 Department of Computer Science, University of New Orleans, New Orleans, LA 70148, USA; fnafees@uno.edu; 5 Alvarado Parkway Institute Behavioral Health System, La Mesa, CA 91942, USA; soroor.heidari@apibhs.com; 6 School of Public Health, Louisiana State University Health Sciences Center-New Orleans (LSUHSC-NO), New Orleans, LA 70112, USA; qyu@lsuhsc.edu; 7 Department of Pathology, Louisiana State University Health Sciences Center-New Orleans (LSUHSC-NO), New Orleans, LA 70112, USA; 8 LSU-LCMC Health Cancer Center, Louisiana State University Health Sciences Center-New Orleans (LSUHSC-NO), New Orleans, LA 70112, USA

**Keywords:** mammary fat pad microenvironment, obesity, Western diet

## Abstract

**Highlights:**

**What are the main findings?**

- The Western Diet significantly contributes to the alteration of cells and tissue morphology.
- The Western Diet significantly modulates the mammary fat pad microenvironment.

**What are the implications of the main findings?**

- The Western Diet induces mammary fat pad inflammation through enhanced immune cell infiltrations.
- Gene expression and cellular processes in the mammary fat pad are significantly influenced by the Western Diet.

**Abstract:**

The mammary gland is a heterogeneous organ that modulates ductal morphogenesis and alveolar differentiation. Obesity is a significant risk factor for several cancers, including postmenopausal breast cancer. We and others have described an association between obesity and increased breast cancer growth. However, the effects of obesity on the mammary fat pad microenvironment (MFPME) remain understudied. Here, we investigated the effect of the Western Diet (WD) on immunocompetent female mice and on their MFPME. Our data suggest that the WD increased body, liver, and perigonadal white adipose tissue (pWAT) weight, as well as myeloid cell infiltration into these tissues. Interestingly, we did not find any significant change in CD4^+^ and CD8^+^ T cells in the liver, blood, and pWAT. NanoString data demonstrates that various cellular processes, including the complement system, innate immune system, phagocytic activity, immune metabolism, and NOD-like receptor (NLR) signaling, were upregulated in the MFPME of obese mice. RNA-Seq data suggest that WD significantly modulated MFPME physiology through regulation of gene expression, cellular processes, and signaling pathways. Further investigation is necessary to determine how WD-mediated changes in MFPME modulate breast cancer biology.

## 1. Introduction

The mammary gland is a heterogeneous organ composed of adipose stromal, fibroblast, epithelial, myoepithelial, immune, and vascular cells. Effective communication and interaction among these cells are necessary for the normal growth, development, and function of the mammary gland [1,2]. Gjorevski et al. summarized that various signaling pathways, including hormone-induced paracrine signaling, tissue geometry, and physical signals, play vital roles in mammary gland development [3]. A unique and important aspect of mammary gland biology is the requirement that mammary epithelial cells grow and function within the stromal environment of the mammary fat pad (MFP) [4]. The MFP plays an essential role in the development of the mammary epithelium by providing cellular signals that are necessary for ductal morphogenesis and alveolar differentiation [4,5]. Cleary et al. showed that consumption of a high-fat diet (HFD) accelerates progression in a transgenic mouse model [6]. Zhao et al. identified that HFD-induced gene expression is associated with malignant cell proliferation, immunosuppression, vascularization, enhanced growth factor production, and reduced tumor latency, among other effects [3,7]. Elliot et al. found that the mammary fat pad microenvironment (MFPME) significantly augments the growth of breast cancer-derived adenocarcinoma cells when transplanted alongside mouse adipose tissue [8]. It is well-established that obesity creates a low-grade chronic inflammatory state, which impairs immune system functions [9,10] and leads to increased risk of developing breast cancer and worse prognosis [11]. One cohort study showed that there is no positive association between dietary fat intake and the risk of breast cancer [12], while another cohort study showed that high BMI is a risk factor for pre- and postmenopausal breast cancer [13]. Increased adiposity is shown to cause chronic macrophage-driven inflammation and adipokine release and alter estrogen and insulin signaling in breast cancer [14]. Another study showed that breast tumors from patients with obesity show higher levels of desmoplasia, which is characterized by increased collagen deposition and alpha-smooth muscle actin-positive fibroblasts [15]. These fibroblasts contribute to fibrosis in the mammary gland and may also play a role in carcinogenesis by altering the tumor microenvironment (TME). Hillers-Ziemer et al. demonstrated that obesity promotes mammary tumor growth by increasing the expression of genes such as *Sox2* and *Notch*, which are associated with cancer stemness as well as by enhancing angiogenesis and macrophage recruitment [16]. Weight loss in women with obesity may lower breast cancer risk by shifting macrophages from a pro-inflammatory to an alternative phenotype [17]. In a diet-induced obese mouse model, Kuziel et al. showed that switching from a high-fat diet to a low-fat diet resolved macrophage- and myeloid cell-mediated inflammation in adipose tissue without altering fibrosis [17]. These findings highlight the role of obesity in establishing an immunosuppressive TME [17]. Further support for this notion comes from a previous study that found that HFD-induced obesity impairs CD8^+^ T cell functions and accelerates tumor growth [18].

In our previous work, we showed that obesity promotes triple-negative breast cancer (TNBC) growth [19]. To further investigate this relationship, in the present study we evaluated how obesity modulates the MFPME and what role it may play in immune regulation. Our results demonstrated that obesity significantly modulated mice’s body and tissue weights, tissue infiltration of different immune cells, and gene expression patterns. WD increased body, liver, and pWAT weights in mice, as well as myeloid cell infiltration into these tissues. Interestingly, WD feeding did not alter CD4^+^ or CD8^+^ T cells in the liver, blood, or pWAT. These findings led us to explore the WD-mediated modulation of MFPME. Our NanoString and RNA seq data demonstrated that for various cellular processes, differentially expressed genes (DEGs) were significantly different between the diet groups. Many of these genes and cellular processes are relevant to the progression of obesity and chronic inflammation. Based on our results, we conclude that obesity modulates MFPME by influencing body weight, fat deposition, immune systems, cellular processes, and signaling pathways.

## 2. Methods and Materials

### 2.1. Mice and Tissues

FVB female mice (4–5 weeks old) were purchased from Jackson Laboratory (Bar Harbor, ME, USA). Following the acclimation period, mice were randomly divided into two groups. Control mice were fed with a regular chow diet (RD) [19% calories from protein, 9% calories from fat, and 44.9% calories from carbohydrates (2019S, ENVIGO, Indianapolis, IN, USA)]. To induce obesity, another group of FVB mice was fed with a Western Diet (WD) [15.2% calories from protein, 42% calories from fat, and 42.7% calories from carbohydrates (TD.88137, ENVIGO, Indianapolis, IN, USA)] for 4 months in our animal care facility. At the end of the experiment, mice were euthanized by carbon dioxide (CO_2_) inhalation, and the whole blood was collected by cardiac puncture. Incision and heart removal were performed to immediately collect various tissues, including perigonadal white adipose tissue (pWAT), liver, MFPs, and adjacent lymph nodes, for subsequent measurements. All animal procedures were approved by the LSUHSC-NO Institutional Animal Care and Use Committee (IACUC).

### 2.2. Tissue Dissociation for Single-Cell Suspension

#### 2.2.1. Blood

Immediately after collecting the whole blood (*n* = 5/group) by cardiac puncture, it was transferred into EDTA tubes (BD Microtainer™ Capillary Blood Collector, Fisher Scientific, Waltham, MA, USA). Blood was transferred into a Falcon 5 mL polypropylene tube (Corning Life Science, Corning, NY, USA) at a ratio of 1:1 with 1X DPBS (Cat# 14190-144, Gibco™ DPBS, Thermo Fisher Scientific, Waltham, MA, USA), centrifuged for 10 min at 800 *g*, and the buffy coat layer residing at the plasma–RBC junction was harvested. Residual RBCs in the buffy coat were lysed using Gibco™ ACK Lysing Buffer (cat# A10492-01, Thermo Fisher Scientific, Waltham, MA, USA), washed, and resuspended in 1X DPBS.

#### 2.2.2. pWAT

Cell suspension from fat tissue (*n* = 5/group) was obtained as described previously [20]. Briefly, both perigonadal fat pads were examined to remove visible blood vessels, weight was recorded, and they were placed on a 60 mm Petri dish containing cold KRBH buffer (10 mM HEPES [pH 7.4], 15 mM NaHCO_3_, 120 mM NaCl, 4 mM KH_2_PO_4_, 1 mM MgSO_4_, 1 mM CaCl_2_, and 2 mM sodium pyruvate) supplemented with 1.5% free-fatty acid BSA (cat# A7030, Sigma-Aldrich, St. Louis, MO, USA) and 2 mM EDTA (cat# AM9260G, Invitrogen, Waltham, MA, USA) to wash and keep the tissue moist. The fat pads were minced into ~5 mm pieces with scalpels and digested with 1.2 mg/mL type II collagenase (cat# C2-BIOC, Sigma-Aldrich, St. Louis, MO, USA) and 10 μg/mL DNase I (CAS 9003-98-9, Roche, Basel, Switzerland) in KRBH buffer for 30 min with gentle rotation (14 rpm) at 37 °C. The tissue was broken into smaller pieces by pipetting at 15 min intervals during incubation. The cell suspension was filtered through a 70 μm cell strainer (Corning, Corning, NY, USA) and centrifuged at 300 *g* for 10 min at 4 °C. Floating adipocytes were removed, and the stromal vascular fraction pellet was resuspended in ACK lysis buffer (cat# A10492-01, Thermo Fisher Scientific, Waltham, MA, USA) to lyse RBCs. The reaction was stopped with Gibco^TM^ DMEM (cat# 11995-065, Thermo Fisher Scientific, Waltham, MA, USA), and cells were centrifuged at 500 *g* for 5 min at 4 °C. The pellet was resuspended in 1× DPBS.

#### 2.2.3. Hepatic Non-Parenchymal Cells (NPCs)

Mouse NPCs were isolated (*n* = 5/group) according to the previously described protocol [20] with minor modifications. Briefly, after recording liver weight, the tissue was placed on a 60 mm Petri dish containing cold KRBH buffer supplemented with 1.5% free-fatty acid BSA and 2 mM EDTA. The liver was minced with a scalpel, and pieces (~3–5 mm) were digested with 1 mg/mL of type IV collagenase (cat# C4-BIOC, Sigma-Aldrich, St. Louis, MO, USA) in the presence of 10 μg/mL DNase I in Gibco^TM^ RPMI-1640 (cat# 11875-093, Thermo Fisher Scientific, Waltham, MA, USA) for 30 min with gentle rotation (14 rpm) at 37 °C. The tissue was broken into smaller pieces by pipetting at the end of the incubation. The cell suspension was filtered through a 70 µm cell strainer and centrifuged at 50 *g* for 3 min at 4 °C to collect the supernatant and remove non-digested and fibrotic tissue that remained in the pellet. NPCs were enriched by density centrifugation using 40 and 60% Percoll (cat# P7828 Sigma-Aldrich, St. Louis, MO, USA) at 325 *g* for 25 min at room temperature. The buffy coat over the 60% Percoll fraction was harvested, washed, and resuspended in 1× DPBS. Red blood cells were lysed using cell lysis buffer, washed, and resuspended in 1× DPBS.

### 2.3. Flow Cytometry

Following incubation with anti-CD16/CD32 antibody to block Fc receptors (cat# 553142, BD Bioscience, Franklin Lakes, NJ, USA), 1 × 10^6^ single cell suspensions were incubated with the following antibodies: Ly6C (cat# 571441, BD Bioscience, Franklin Lakes, NJ, USA), CD45 (cat# 56-0451-82, eBioscience, San Diego, CA, USA), CD11b (cat# 571486, BD Biosciences, Franklin Lakes, NJ, USA), Ly6G (cat# 571207, BD Biosciences, Franklin Lakes, NJ, USA), Gr1 (cat# 563849, BD Biosciences, Franklin Lakes, NJ, USA), and F4/80 (cat# 569224, BD Biosciences, Franklin Lakes, NJ, USA), for 20 min. Cells were washed once with 2 mL 1× DPBS and then resuspended in 200 μL 1× DPBS for analysis using the Gallios Flow Cytometer configured with 3 lasers and 8 colors (Beckman Coulter, Brea, CA, USA). Sequential flow cytometry gating was performed to exclude debris, doublets, and dead cells. The target cell population was then analyzed for CD45^+^ expression, followed by cell-type-specific gating as mentioned in the figure legends. For flow cytometry analysis, 5 samples per group were used, and all samples were run with the same voltage settings. Data was analyzed using Kaluza software v2.1(Beckman Coulter, Brea, CA, USA).

### 2.4. Histology and Immunohistochemistry

Mouse MFP and adjacent lymph node tissues were fixed in 10% buffered formalin for tissue processing. After paraffin embedding, tissues were sectioned at a thickness of 4 μm, placed on electromagnetically charged slides (Fisher Scientific, Waltham, MA, USA), and stained with Hematoxylin & Eosin (H&E) for routine histological analysis. Immunohistochemistry was performed using the Avidin–Biotin–Peroxidase complex system, according to the manufacturer’s instructions (cat# PK-6100, Vectastain Elite ABC Peroxidase Kit; Vector Laboratories, Burlingame, CA, USA). Our modified protocol includes melting the paraffin at 56 °C for 15 min, clearing in xylenes 3 times for 15 min each, rehydration through descending grades of alcohol up to water, non-enzymatic antigen retrieval with 0.01 M sodium citrate buffer pH 6.0 at 95 °C for 25 min in a vacuum oven, and endogenous peroxidase quenching with 3% H_2_O_2_ in methanol; following these steps, slides were washed with PBS and blocked in PBS/0.1% BSA containing 5% normal horse serum (for mouse and rat monoclonal antibodies) for 2 h at room temperature, then incubated overnight with primary antibodies. Primary antibodies included a rat monoclonal anti-CD11b (Clone M1/70, cat# 101212, Bio Legend, San Diego, CA, USA, dilution 1:50), a rabbit recombinant monoclonal anti-CD8 (cat# ab217344, Abcam, EPR21769, Abcam, Cambridge, UK, dilution 1:2000), a rabbit monoclonal anti-F4/80 (cat# ab240946, Clone SP115, Abcam, Cambridge, UK, dilution 1:100), and a rat monoclonal anti-FOXP3 (cat# 17-5773-82, Clone FJK-16s, Invitrogen, Waltham, MA, USA, dilution 1:100). After overnight incubation, slides were rinsed thoroughly with PBS and incubated with biotinylated anti-rabbit or anti-rat secondary antibodies for 1 h at room temperature. Then, Avidin–Biotin complexes were incubated for 1 h at room temperature, and finally, slides were developed using a diaminobenzidine substrate DAB (cat# 05269806001, Roche Laboratories, Basel, Switzerland), counterstained with Hematoxylin, and mounted with Permount. Images were collected using a BX61 Olympus microscope equipped with a high-resolution Olympus DP80 digital camera and CellSens image capture software (v3.4) (Evident Scientific, Waltham, MA, USA).

### 2.5. RNA Extraction from Mouse MFP

Total RNA was extracted from fresh snap frozen adipose tissue samples (*n* = 4/group) using the RNeasy lipid tissue mini kit (cat# 74804, Qiagen, Hilden, Germany). Directions from the manufacturer were followed. Briefly, frozen tissues (50–70 mg) were first disrupted and homogenized by bead-milling using 2.8 mm ceramic beads (cat# 14-828-348, Fisher Scientific, Waltham, MA, USA) and the Omni Bead Ruptor 4 (Omni International, Kennesaw, GA, USA), followed by 5 min incubation at room temperature. Homogenization was followed by the addition of chloroform, vigorous shaking, and centrifugation. The resulting upper aqueous phase was next purified using the RNA purification columns as indicated by the manufacturer’s instructions. A DNase I treatment (cat# 79254, Qiagen, Hilden, Germany) was included. RNA was eluted in 30 μL RNase-free H_2_O and quantified using the Qubit RNA HS Assay kit (cat# Q32852, Invitrogen, Waltham, MA, USA). RNA quality was assessed with an Agilent 2100 bioanalyzer (Agilent Technologies, Santa Clara, CA, USA).

### 2.6. RNA Library Preparation, Sequencing, and Analysis

RNA quantification was performed using the Qubit RNA Broad Range Assay kit (cat# Q32852, Invitrogen, Waltham, MA, USA), and RNA quality was assessed with the Agilent 2100 bioanalyzer (Agilent Technologies, Santa Clara, CA, USA). Libraries were generated using Illumina Stranded Total RNA prep with Ribo-Zero Plus library preparation kit (cat# 20040526, Illumina, San Diego, CA, USA) according to the manufacturer’s instructions. Briefly, ribosomal RNA was first depleted from total RNA (100 ng) with Ribo-Zero plus kit (cat# 20037135, Illumina, San Diego, CA, USA). Following purification, the RNA was fragmented, and first and second-strand cDNA was synthesized. Libraries were created from the cDNA by first adenylating the 3′ end and ligating anchors to the ends. An amplification step of 13 cycles of PCR was then performed to add unique indexes to the ligated products. Resulting libraries were quantified using the Qubit dsDNA High Sensitivity Assay Kit (cat# Q33231, Invitrogen, Waltham, MA, USA), and size and purity were assessed with the Agilent 2100 bioanalyzer (Santa Clara, CA, USA). Sequencing was performed on Illumina’s Next Seq 2000 instrument (Illumina, San Diego, CA, USA) using a Next Seq 2000 P2 200-cycle kit (cat# 20100981, Illumina, San Diego, CA, USA) with pair-end 2 × 76 bp reads. FASTQ files were downloaded from the Illumina Base Space Hub and uploaded to Partek Flow (v12.9.1), and reads from contaminants (rDNA, tRNA, mtrDNA) were removed with Bowtie 2 (v2.2.5). Reads were aligned to STAR v2.7.8a and quantified with Ensembl v102 using the mm10 as the reference genome. Features (genes) with fewer than 5 reads were excluded from downstream analyses. Differential gene expression between obese and control mice (*n* = 4/group) was assessed using DESEQ2 with TMM-normalized counts (+0.0001/TMM/log2). Adjustment for multiple comparisons was carried out at the False Discovery Rate (FDR) ≤ 0.05. Pathway analysis (KEGG) and gene ontology (GO) term analysis were performed with a significance cut-off of FDR ≤ 0.05.

### 2.7. Gene Expression Analysis by NanoString

Total RNA from the mouse MFP (*n* = 4/group) was used to detect 579 immune-related genes using the nCounter Immunology panel, following the manufacturer’s instructions (NanoString, Seattle, WA, USA). Briefly, 75 ng of RNA was hybridized for 16 h using capture and reporter probes. Detection was performed in the nCounter SPRINT system (NanoString, Seattle, WA, USA). Data analysis was conducted using nSolver software v4.0 (NanoString, Seattle, WA, USA), including the nCounter Advanced Analysis add-on software (v2.0).

### 2.8. Statistical Analysis of Cell Subpopulations from Tissues

The *t*-test statistical analysis was used to determine whether significant differences exist between the means of the two groups, RD and WD. A *p*-value of ≤0.05 was considered statistically significant. GraphPad Prism 10.6.0 was used for graphing and data analysis.

## 3. Results

### 3.1. Western Diet Increased Body Weight, Tissue Weight, and Immune Cell Populations in Mice

**Figure 1:**
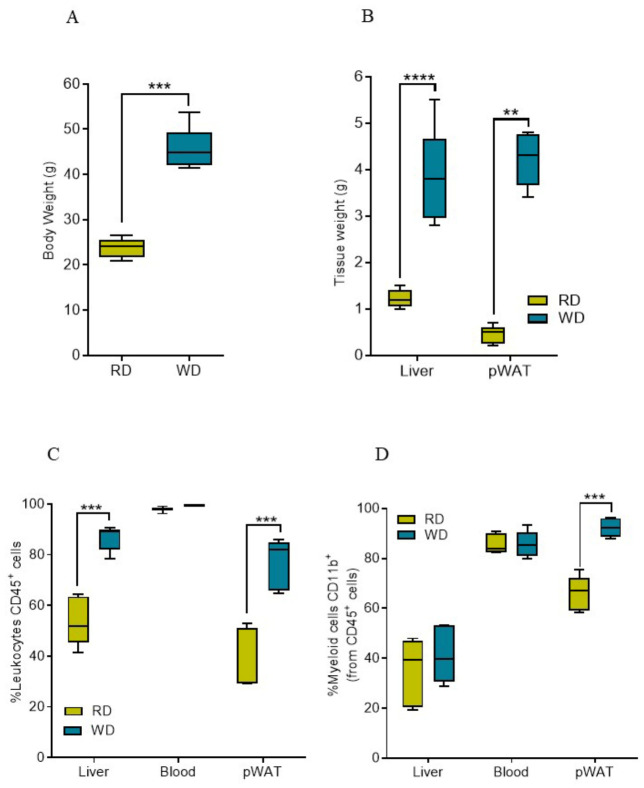
The Western Diet (WD) significantly increases body weight, tissue weight, and immune cell populations in mice. Mice were fed either a regular diet (RD) or a Western Diet (WD) for 4 months (*n* = 5/group). (**A**) Body weight was measured twice per week. (**B**) After sacrifice, liver and perigonadal white adipose tissue (pWAT) weights were measured. (**C**) Percentage of CD45^+^ leukocytes in liver, blood, and pWAT as measured by flow cytometer. (**D**) Percentage of CD11b^+^ myeloid cells within CD45^+^ leukocytes, assessed by flow cytometry. Data are presented as mean ± SEM. Statistical significance was determined using Student’s *t*-tests or one-way ANOVA in GraphPad Prism: ** *p* < 0.01, *** *p* < 0.001, **** *p* < 0.0001.

To confirm WD-induced obesity, we measured body and tissue weights and found that WD significantly increased body weight relative to RD (Figure 1A).

As expected, WD increased both liver and pWAT weights (Figure 1B), suggesting adipose tissue hypertrophy and liver steatosis. CD45, also known as leukocyte common antigen, is a receptor-associated protein tyrosine phosphatase that is expressed on all leukocytes and plays an important role in intracellular signaling pathways [21]. Researchers have demonstrated that infiltrating CD45^+^ cells are increased in adipose tissue during obesity, indicating an inflamed tissue [22]. Our results also showed that total leukocyte counts were significantly higher in both liver and pWAT derived from WD-fed mice (Figure 1C). We then examined the marker CD11b, a cell-surface integrin expressed on myeloid cells, such as macrophages, granulocytes, and NK cells [23]. Although WD did not affect the percentage of CD45^+^CD11b^+^ myeloid cell populations in the liver and blood, pWAT showed a significant increase in WD-fed mice compared to the RD group. Taken together, these data confirm the previous reports [24,25] demonstrating that WD induced hypertrophy in pWAT weights, which is largely associated with the infiltration of myeloid cells.

### 3.2. Western Diet Altered Myeloid-Derived Suppressor Cells (MDSCs) and Macrophage Populations in Tissues

**Figure 2:**
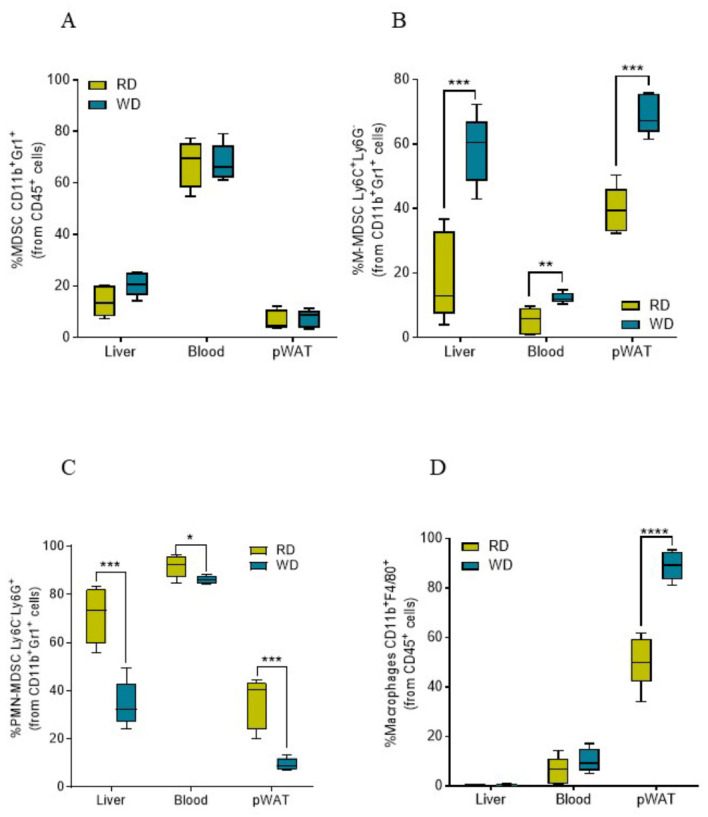
Western Diet alters myeloid-derived suppressor cell (MDSC) and macrophage populations in tissues. Mice were fed a regular diet (RD) or a Western Diet (WD) for 4 months (*n* = 5/group), and immune cell subsets were quantified by flow cytometry. (**A**) Percentage of total MDSCs (CD11b^+^Gr1^+^) among CD45^+^ leukocytes in liver, blood, and perigonadal white adipose tissue (pWAT). (**B**) Percentage of monocytic-MDSCs (M-MDSCs, CD11b^+^Gr1^+^Ly6C^+^Ly6G^−^), (**C**) percentage of polymorphonuclear-MDSCs (PMN-MDSCs, CD11b^+^Gr1^+^Ly6C^−^Ly6G^+^). (**D**) Percentage of macrophages (CD11b^+^F4/80^+^) from CD45^+^ leukocytes. Data analyses were performed in GraphPad Prism, and statistical significance was determined using one-way ANOVA, with * *p* < 0.05, ** *p* < 0.01,*** *p* < 0.001, and **** *p* < 0.0001.

MDSCs are immature immune cells that expand during chronic inflammation, including obesity, and have potent immunosuppressive activity [26,27]. Based on the expression of cell surface receptors, MDSCs can be divided into two distinct subgroups: monocytic MDSCs (M-MDSCs), which express Ly6C, and polymorphonuclear MDSCs (PMN-MDSCs), which express Ly6G as well [28,29]. Our results showed that the overall percentage of CD11b^+^ Gr1^+^ MDSCs across tissues did not differ significantly between RD and WD (Figure 2A), not because the subsets remained unchanged, but because of the opposite effect of WD on the MDSC subtypes.

Specifically, WD markedly increased tissue infiltration of M-MDSCs (Figure 2B), while PMN-MDSCs were reduced to a similar extent (Figure 2C). The percentage of CD11b^+^F4/80^+^ macrophages derived from CD45^+^ cells was significantly increased only in pWAT in the WD group (Figure 2D). Our results suggest that WD significantly induced the tissue infiltration of M-MDSCs while macrophages were specifically recruited into the inflamed adipose tissue.

### 3.3. T Lymphocytes Were Unaltered in the WD Group

**Figure 3:**
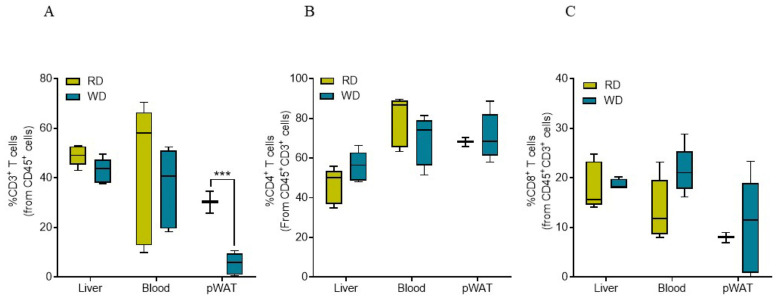
T lymphocytes are mostly unchanged in the WD group. Mice were fed a regular diet (RD) or a Western Diet (WD) (*n* = 5/group) for 4 months, and immune cell subsets were quantified by flow cytometry. (**A**) Percentage of CD3^+^ cells in liver, blood, and pWAT. Only CD3^+^ T cells were downregulated in pWAT of WD-fed mice. (**B**) The percentage of CD4^+^ T cells was unaltered in the liver, blood, pWAT. (**C**) The percentage of CD8^+^ T cells was also unchanged in the liver, blood, and pWAT between the groups. Data analyses were performed using GraphPad Prism, and statistical significance was determined using one-way ANOVA, with *** *p* < 0.001.

T cells are known to play a crucial role in obesity and chronic inflammatory diseases [30,31,32]. To evaluate the impact of obesity on T lymphocytes, we quantified the frequencies of CD3^+^, CD4^+^, and CD8^+^ T cells infiltrating the liver, blood, and pWAT. Although total CD3^+^ T cells were significantly altered by WD in pWAT and remained unchanged in the liver and blood (Figure 3A), no significant differences were observed within the CD4^+^ and CD8^+^ subsets individually (Figure 3B,C), suggesting that the overall change reflects a combined or modest shift across T subpopulations cell rather than a strong effect on a specific subset.

### 3.4. Tissue Infiltration of Immune Cells Was Increased in the Lymph Nodes of Obese Mice

**Figure 4:**
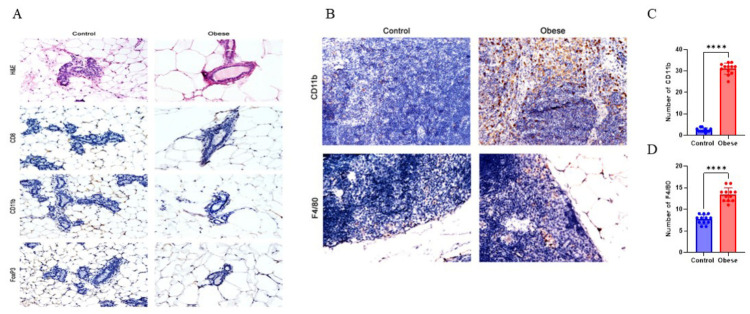
Immune cell infiltration in the MFP and lymph node. Panel (**A**) represents H&E and immunohistochemical labeling of breast tissue for CD8, CD11b, and FoxP3. (**B**) Immunohistochemical labeling of the lymph node for CD11b and F4/80. (**C**,**D**) Quantification of CD11b^+^ and F4/80^+^ cells, respectively. Data are presented as mean ± SEM. Statistical significance was determined using Student’s *t*-tests in GraphPad Prism: **** *p* < 0.0001.

Obesity induces inflammation in the mammary adipose tissue, characterized by crown-like structures, along with increased macrophage infiltration [33]. Obesity is also associated with perilymphatic inflammation, where a structurally and functionally compromised lymphatic system becomes immunologically less optimal [34,35]. Therefore, we compared immune cells in the MFP with those in its adjacent lymph node. We performed H&E and immunohistochemistry for CD8, CD11b, F4/80, and FoxP3, a marker of T regulatory cells, in the MFP of lean and obese mice. We found that adipocyte size in MFP was larger in the obese group than in the control group; however, the infiltration of CD8, CD11b, and FoxP3-positive cells was not different in MFP between the groups (Figure 4A).

Next, we performed immunohistochemistry on the MFP-adjacent lymph node for CD11b and macrophages (F4/80). The results demonstrated that both CD11b^+^ myeloid cells and macrophages (F4/80^+^) were significantly enriched in WD-fed mice (Figure 4B). Our findings indicate that WD markedly enhances the infiltration of myeloid cells, including macrophages, in the lymph nodes.

### 3.5. Cellular Processes and Pathways Were Significantly Different in WD-Induced Obese Mouse MFP

**Figure 5:**
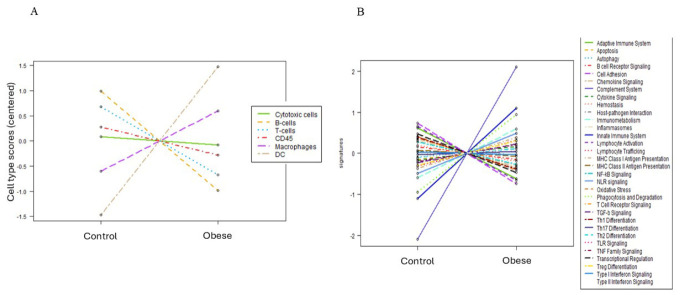
NanoString nCounter analysis of differential gene expression in MFP of control and obese mice. NanoString nCounter analysis was performed to quantify the expression of immune-related genes in control vs. obese groups (*n* = 4/group). (**A**) Cell type analysis between the control and obese groups. (**B**) Adaptive and innate immune system pathways within the MFP.

Different cellular pathways and processes are known to be involved in the development of obesity [36]. Hence, we performed NanoString analysis and RNA sequencing to assess differential gene expression (DGE) and signaling pathway enrichment in MFP from obese and control mice. Our NanoString results demonstrated that infiltration of different cell types was differently modulated in obese groups. The infiltration of dendritic cells (DCs) and macrophages was significantly upregulated in the obese group, whereas B-cells, T cells, and CD45^+^ cells were significantly downregulated (Figure 5A).

**Figure 6:**
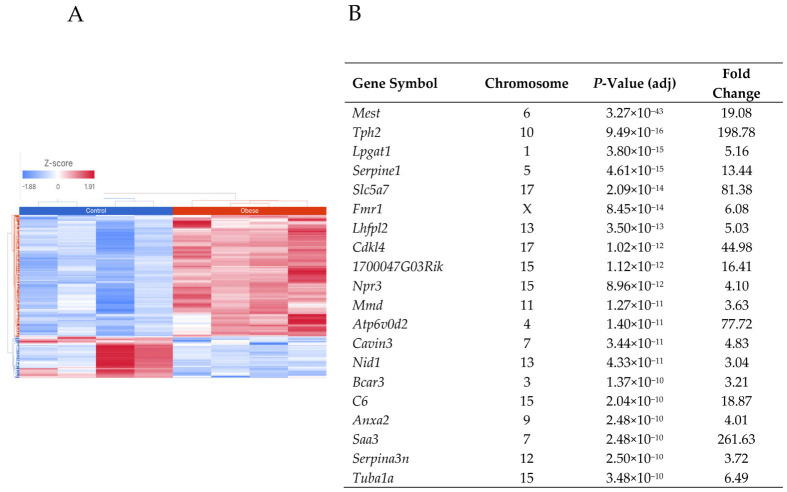
Differential gene expressions between the control and obese mouse MFP. (**A**) Hierarchical clustering of expression changes seen by RNA-seq between two groups (*n* = 4/group). Expression values were presented as z-scores, with red indicating higher expression and blue indicating lower expression. (**B**) Top 20 differentially expressed genes (DEGs) between control and obese mice.

Our results also demonstrated that cellular signaling pathways were significantly altered in the obese group compared with the control group. Namely, the complement system, innate immune system, phagocytic activity, immune metabolism, and NLR signaling, among others, were upregulated in obese mice (Figure 5B). On the other hand, different cellular signaling pathways were downregulated in obese mice, such as lymphocyte activation, adaptive immune system, transcriptional regulation, and Th1 differentiation (Figure 5B). Through our RNA sequencing analysis, we found that 1052 genes were differentially expressed, with statistically significant differences (FDR ≤ 0.05) between the groups (Figure 6A and Table S1).

Figure 6B presents the top 20 DEGs in order of significance. Notably, mesoderm-specific transcript (*Mest*), tryptophan hydroxylase 2 (*Tph2*), lysophosphatidylglycerol acyltransferase 1 (*Lpgat1*), serpin family E member 1 (*Serpine1*), and solute carrier family 5 member 7 (*Slc5a7*) were the top five differentially expressed genes in the obese group. Using the RNA sequencing data, we performed pathway enrichment analysis. We observed that 24 pathways were significantly different (FDR ≤ 0.05) between the groups (Table S2). Among those, the top 20 pathways are presented in Table 1.

The most upregulated pathways are related to lysosome, complement and coagulation cascades, hematopoietic cell lineage, phagosome, and PPAR signaling pathway. We performed Gene Ontology (GO) analysis and identified 1312 significantly different cellular processes (FDR ≤ 0.05) between the obese and control groups (Table S3). Table 2 lists the top 20 cellular functions and biological processes that were different, including membrane, protein binding, membrane part, collagen-containing extracellular matrix, and plasma membrane.

Next, we aim to cross-validate the DEGs with an independent dataset. We explored the GEO database (number GSE42840) and found that the control and obese mice had 719 differentially expressed genes. We found 51 common genes among our RNA-seq data *(n* = 1052), NanoString data (*n* = 68), and GEO42840 (*n* = 719) (Figure S1). We input those genes into DAVID (https://davidbioinformatics.nih.gov/workspace.html?tool=summary_ws) (accession date: 25 May 2026) and found that those 51 genes were involved in pathways associated with metabolism (cholesterol metabolism, lysosome biogenesis, efferocytosis, and fat digestion and absorption). Furthermore, NanoString used an analysis limited to nearly 579 genes (mouse panel), whereas with RNA-seq, we were able to interrogate the whole transcriptome. However, 21 of the 68 DEGs identified by NanoString were also present in the list of 1052 DEGs identified by RNA-seq (Figure S2). Despite differences in DEGs, both algorithms (SDV and GSA by NanoString and KEGG) identified similar pathways in which these genes are involved. Taken together, our results suggest that WD-induced obese mice have differentially expressed genes, signaling pathways, and cellular processes in the MFP.

## 4. Discussion

Obesity has emerged as a major global public health burden and continues to rise at an alarming rate, despite increased awareness and prevention efforts. A typical WD, characterized by high calories, saturated fat, and sugar, contributes significantly to obesity [37,38]. Our results showed a similar trend in body weight gain when mice were fed with WD compared with RD. We also observed significant increases in liver and pWAT weights, which play important roles in fat regulation and metabolism. Our observations are supported by another study that found that mice fed with WD for 22 weeks gained 92% more weight than those on RD [39]. Low-grade inflammation is observed in obesity due to activation of certain cellular mechanisms. Overfeeding can stimulate inflammation in metabolism-related tissues such as adipose tissue, liver, and muscle [40]. A previous study in humans revealed that in the setting of obesity, the numbers of leukocytes, lymphocytes, CD4^+^, and CD8^+^ cells are significantly increased, and a state of chronic inflammation is induced by cytokine release from adipose tissue [41,42]. Our results showed that the number of leukocytes increased significantly in the liver and pWAT, and that the number of myeloid cells increased significantly in pWAT from obese mice. Another study also found increased levels of PMN-MDSCs in an obesity-induced cancer model [43]. Here, we used a WD-fed non-tumor model to investigate the role of MDSCs in chronic inflammation and made several observations. We did not find any overall changes in MDSCs percentages in the liver, blood, and pWAT of WD-fed mice. While the number of M-MDSCs increased significantly in the obese group, the number of PMN-MDSCs decreased significantly. Since we used a non-tumor model, this might explain the difference in MDSC subsets in our model.

A previous study demonstrated that CD4^+^ T cells and TAMs, such as CD11b^+^ F4/80^+^ cells, are responsible for MFP inflammation [44]. In our study, we did not find any significant differences in CD4^+^ and CD8^+^ T cell populations between the treatment groups. Another study demonstrated that Th17 cells are increased in HFD-induced obese mice and play a pivotal role in inducing inflammation in adipose tissue, whereas Treg cells are decreased under obese conditions and normally suppress inflammation [45]. This balance between Th17/Treg cells in obese conditions helps explain why no changes were observed in the CD4^+^ T cell population across tissues between treatments. In our study, we observed that the CD11b^+^ F4/80^+^ cells were increased in pWAT derived from WD-fed mice. Taken together, these findings further highlight the important role obesity plays in inducing MFP inflammation. We then examined the lymph node, a specialized organ responsible for the surveillance of immune responses [46]. In the lymph node, we observed markedly increased infiltration of immune cells in obese mice (myeloid cells and macrophages) compared to the control group. This result is consistent with previous findings that examined the effects of HFD on changes in visceral lymph node immune cell populations [47,48].

Our NanoString data demonstrated that various cellular processes, including the complement system, innate immune system, phagocytic activity, immune metabolism, and NLR signaling, were upregulated in obese mice. Previously, it has been reported that the complement system and innate immune system link obesity and inflammatory diseases [49,50]. In obese individuals, innate immune cells, such as macrophages, neutrophils, and dendritic cells, accumulate in adipose tissue and lead to chronic low-grade inflammation through the production of pro-inflammatory cytokines and chemokines [51]. This is consistent with our analysis in obese mice, which showed upregulation of the innate immune system and infiltration by macrophages and dendritic cells. The complement system, best known for its role in fighting infections, also plays an important role in obesity. Fat tissue can produce complement proteins such as C3 and B, which not only induce fat accumulation but also trigger low-grade inflammation as fat mass expands [52]. In people with obesity, the constant activation of the complement system has been linked to insulin resistance, chronic inflammation, and a higher risk of metabolic syndrome [53]. Phagocytic activity and immune metabolism are also upregulated in obese mice, serving as hallmarks of the low-grade inflammatory state associated with obesity [54,55,56,57]. In obesity, adipocytes become stressed and damaged, undergoing apoptosis. Macrophages enhance their phagocytic activity to remove these apoptotic cells and transform into foam cells, which can exacerbate insulin resistance and inflammation through a shift in phenotype and function [25,58,59]. Our results support other previous findings regarding the correlation between obesity and inflammation [60,61]. NanoString data analysis also revealed that NLR signaling was upregulated in obese mice. NLRs are a family of cytosolic pattern recognition receptors that play an important role in sensing obesity-associated danger signals and inducing low-grade inflammatory responses that link adiposity to metabolic dysfunctions such as insulin resistance, type 2 diabetes, atherosclerosis, and fatty liver disease [51,62]. Researchers have identified that the upregulated pathways in NLR signaling, NLRP3, contribute greatly to inflammatory diseases in obese mice [63,64,65].

Our RNA-seq data showed DEGs between the groups. Many of these genes are relevant to the progression of obesity and chronic inflammatory disease states. *Mest*, *Tph2*, *Lpgat1*, *Serpine1*, and *Slc5a7* were all upregulated in obese mice. Among those genes, *Mest* is associated with adipocyte development and obesity [66]. The *Mest* gene encodes a protein with enzymatic function [67] and is closely associated with increased adipocyte size and fat mass expansion [68]. On the other hand, mice lacking functional *Mest* show reduced diet-induced adipose tissue growth, smaller fat cells, and improved glucose tolerance that are associated with reduced inflammation [69]. Researchers have also demonstrated that *Mest* exacerbates obesity-associated inflammation and metabolic dysfunction [70]. Our DEG analyses also demonstrated the upregulation of the *Tph2* gene in the obese group. *Tph2* is responsible for the synthesis of serotonin in the brain [71]. Serotonin (5-HT) is known to play a dual role in the nervous system. The serotonin signal in the central nervous system is associated with anorexia and high energy expenditure, whereas the peripheral serotonin signal promotes energy storage and fat accumulation, thereby contributing to obesity [72]. Researchers have shown that obesity is associated with a marked increase in adipocyte-specific *Tph2* expression in both obese mice and humans, contributing to elevated local and circulating serotonin levels that impair brown adipose tissue (BAT) thermogenesis and exacerbate metabolic dysfunction [73]. These findings are consistent with our results, which were derived from diet-induced obese mice.

*Lpgat1* was upregulated in obese mice and is an endoplasmic reticulum-localized acyltransferase that catalyzes the reacylation of lysophosphatidylglycerol to phosphatidylglycerol [74]. *Lpgat1* plays an important role in maintaining mitochondrial membrane lipid composition and has been implicated in metabolic regulation [75]. Researchers have identified that this gene is upregulated in obese mice and plays a crucial role in lipid remodeling within cellular membranes [76]. Interestingly, a previous study has shown that a genetic variant of this gene is strongly associated with higher BMI and body fat percentage in certain populations, suggesting a role in human susceptibility to obesity [77]. *Serpine1*, a family member of serine proteinase inhibitor also known as plasminogen activator inhibitor-1 (PAI-1), is often elevated in obesity [78,79]. Elevated PAI-1 levels impair fibrinolysis and are closely linked to insulin resistance, fatty liver, and abnormal lipid metabolism [80]. Researchers have demonstrated that deficiency or pharmacologic inhibition of PAI-1 protects against obesity-related complications, such as glucose intolerance and hepatic steatosis, indicating its role in driving metabolic dysfunction [81,82,83,84,85]. These findings indicate that *Serpine1* plays an important role in the link between obesity and metabolic diseases.

The pathway enrichment analysis demonstrated that several biological pathways and functions were significantly modulated in the obese group. The top pathways and/or cellular functions are the lysosome, complement and coagulation cascades, the hematopoietic cell lineage, the phagosome, and the PPAR signaling pathway. Lysosomes are cytoplasmic organelles containing many hydrolytic enzymes responsible for the degradation and recycling of macromolecules and the regulation of metabolic homeostasis. These organelles are increasingly recognized as central regulators of metabolic homeostasis, including lipid metabolism and autophagy [86]. The hematopoietic cell lineage pathway regulates the development of blood and immune cells, such as macrophages, B cells, and T cells. In obesity, hematopoietic progenitor cells are reprogrammed to produce myeloid cells, including monocytes and macrophages [65,87]. These cells infiltrate adipose tissue and produce chronic low-grade inflammation, insulin resistance, and adipocyte dysfunction [87]. Studies have demonstrated that classical and alternative complement pathways are upregulated in adipose tissue during obesity, promoting the recruitment and activation of macrophages and propagation of pro-inflammatory signaling and increased local and systemic inflammation [88]. It is also well established that chronic inflammation in adipose tissue can activate coagulation cascades through the action of inflammatory cytokines [89]. Our study also supports these findings, as we observed that numerous inflammation-related molecules and signaling pathways were significantly upregulated in the obese group.

Phagosomes are dynamic organelles in macrophages involved in clearing tissue debris, remodeling tissue, and preventing the spread of pathogens. Notably, phagosome activity was upregulated in obese mice compared to controls [90]. Obesity is characterized by increased adipocyte stress and cell death, which leads to enhanced recruitment and activation of macrophages in adipose tissue. These infiltrated macrophages rely on phagosome formation to clear apoptotic adipocytes and lipid debris [25,59]. Our results also showed similar patterns of phagosome pathway upregulation in obese mice, suggesting how altered phagosome-related pathways link innate immune dysfunction with chronic inflammation in obesity. Our analysis also revealed that peroxisome proliferator-activated receptor (PPAR) signaling was upregulated in obese mice. Activation of these receptors influences different cellular activities, including insulin sensitization, energy homeostasis, and fatty acid metabolism [91,92]. Researchers have identified that impaired PPAR signaling is a key molecular mechanism linking obesity to insulin resistance and related metabolic disorders [93]. Our findings support prior reports, as PPAR was upregulated in obese mice, indicating its role in counteracting obesity-induced inflammation.

We also performed GO analysis and found the upregulation of several cellular processes and locations in obese mice. The following cellular processes and locations were significantly modulated: membrane, protein binding, membrane part, collagen-containing ECM, and plasma membrane. In obesity, adipose tissue exhibits increased expression of genes associated with the plasma membrane, including receptors, transporters, and adhesion molecules. These changes enhance nutrient sensing, intracellular signaling, and immune cell interactions, facilitating the recruitment and activation of immune cells in expanding fat depots [94,95]. Additionally, researchers have demonstrated that protein binding dynamics are altered in obesity due to changes in post-translational modifications of plasma proteins. These changes can further modulate physiological and cellular activities such as inflammation, insulin resistance, and systemic metabolic dysregulation [96,97,98]. Our GO analysis showed higher protein binding activity in the obese group, consistent with previous findings. Moreover, it has been noted previously that obesity is associated with increased expression of genes encoding components of the collagen-containing ECM. Excessive collagen deposition and ECM remodeling lead to adipose tissue fibrosis, impaired adipocyte expansion, and tissue stiffening, which collectively worsen insulin resistance and overall metabolic health [99,100,101]. A previous study had demonstrated that obesity contributes to tumor progression through complex interactions within the TME [18]. Recently, it has also been reported that adipose tissue-derived cytokines, adipokines, and growth factors are responsible for activating oncogenic signaling pathways for cell proliferation, angiogenesis, metastasis, and therapy resistance [102,103].

Although our study demonstrates that obesity modulates MFPME through immune cell infiltration and the modulation of gene expression, pathways, and cellular processes, some limitations should be acknowledged. Primarily, this study was conducted using a non-tumor model, which does not fully support the relationship between obesity and cancer. Further, functional characterization of immune cells in MFPME, such as activation, inactivation, or exhaustion, as well as mechanistic relationships among gene expression, pathways, cellular functions, and obesity, warrants exploration.

## 5. Conclusions

Based on our results, we conclude that obesity modulates MFPME by influencing body weight, fat deposition, immune systems, cellular processes, and signaling pathways. Studies have demonstrated that the interactions of cancer cells with the TME and reprogramming of adipocytes to cancer-associated adipocytes (CAA) enhance cancer progression by providing a favorable tumor-promoting microenvironment [104,105]. We have also previously shown that obesity positively modulates TNBC growth [19]; however, further investigations are required to elucidate the mechanisms by which MFPME modulation influences breast cancer initiation, development, and progression.

## Figures and Tables

**Table 1:** Pathway enrichment analysis of RNA-Seq data. Analysis was performed using differentially expressed genes (DEGs) identified from control and obese RNA-seq data (*n* = 4/group). The top 20 significantly enriched pathways are listed in the table.

Pathway Name	Enrichment Score	FDR
Lysosome	29.01	8.60 × 10^−11^
Complement and coagulation cascades	20.12	3.15 × 10^−7^
Hematopoietic cell lineage	16.37	8.92 × 10^−6^
Phagosome	14.88	2.97 × 10^−5^
PPAR signaling pathway	12.35	2.97 × 10^−4^
Cholesterol metabolism	11.85	4.07 × 10^−4^
ECM−receptor interaction	11.56	4.68 × 10^−4^
Biosynthesis of unsaturated fatty acids	10.85	8.30 × 10^−4^
T cell receptor signaling pathway	10.57	9.75 × 10^−4^
Primary immunodeficiency	10.32	1.13 × 10^−3^
Cell adhesion molecules	9.40	2.57 × 10^−3^
Rheumatoid arthritis	8.99	3.58 × 10^−3^
Focal adhesion	8.86	3.73 × 10^−3^
PD−L1 expression and PD−1 checkpoint pathway in cancer	8.59	4.54 × 10^−3^
Fatty acid metabolism	8.10	6.96 × 10^−3^
Ferroptosis	7.50	1.18 × 10^−2^
Protein digestion and absorption	7.37	1.27 × 10^−2^
Cytokine−cytokine receptor interaction	6.94	1.84 × 10^−2^
NF−kappa B signaling pathway	6.70	2.22 × 10^−2^
Regulation of lipolysis in adipocytes	6.54	2.47 × 10^−2^

**Table 2:** Gene Ontology (GO) enrichment analysis of differentially expressed genes (DEGs). GO analysis was performed based on the DEG derived from RNA-seq data of control and obese mice (n = 4/group). The top 20 enriched GO terms are listed in the table.

Description	Enrichment Score	FDR
Membrane	174.539	3.47 × 10^−72^
Protein binding	111.871	2.86 × 10^−45^
Membrane part	99.5282	4.37 × 10^−40^
Collagen−containing extracellular matrix	94.4829	5.09 × 10^−38^
Plasma membrane	94.1257	5.82 × 10^−38^
Binding	89.2221	6.53 × 10^−36^
Extracellular region part	88.3775	1.30 × 10^−35^
Extracellular matrix	85.9998	1.23 × 10^−34^
Regulation of response to stimulus	83.6582	1.14 × 10^−33^
Plasma membrane part	80.2637	3.05 × 10^−32^
Positive regulation of biological process	79.9274	3.88 × 10^−32^
Extracellular space	79.7733	4.15 × 10^−32^
Regulation of localization	78.9429	8.78 × 10^−32^
Regulation of multicellular organismal process	70.6604	3.04 × 10^−28^
Cell surface	70.651	3.04 × 10^−28^
Regulation of biological quality	69.236	1.17 × 10^−27^
Cell part	66.2995	2.08 × 10^−26^
Regulation of immune system process	65.3452	5.10 × 10^−26^
Intrinsic component of membrane	64.9893	6.90 × 10^−26^
Regulation of locomotion	64.0376	1.70 × 10^−25^

## Data Availability

The original contributions presented in this study are included in the article/Supplementary Materials. Further inquiries can be directed at the corresponding author.

## Supplementary Materials

The following supporting information can be downloaded at: https://www.mdpi.com/article/10.3390/cells15121050/s1, Figure S1: NanoString, RNA-seq, and GEO database (number GSE42840) integration between control and obese groups (*n* = 4/group); Figure S2: NanoString and RNA-seq data integration between control and obese groups (*n* = 4/group); Table S1: Genes were differentially expressed (DE) between the control and obese mouse groups; Table S2: Pathway enrichment analysis of control and obese mice. Analysis was performed based on the differentially expressed genes (DEGs) derived from control and obese RNA-seq data; Table S3: Gene Ontology (GO) enrichment analysis of differentially expressed genes (DEGs). GO analysis was performed based on the DEG derived from RNA-seq data of control and obese mice.

## References

[1] Polyak K., Kalluri R. (2010). The role of the microenvironment in mammary gland development and cancer. Cold Spring Harb. Perspect. Biol..

[2] Macias H., Hinck L. (2012). Mammary gland development. Wiley Interdiscip. Rev. Dev. Biol..

[3] Gjorevski N., Nelson C.M. (2011). Integrated morphodynamic signalling of the mammary gland. Nat. Rev. Mol. Cell Biol..

[4] Neville M.C., Medina D., Monks J., Hovey R.C. (1998). The mammary fat pad. J. Mammary Gland. Biol. Neoplasia.

[5] Couldrey C., Moitra J., Vinson C., Anver M., Nagashima K., Green J. (2002). Adipose tissue: A vital in vivo role in mammary gland development but not differentiation. Dev. Dyn..

[6] Cleary M.P., Grande J.P., Maihle N.J. (2004). Effect of high fat diet on body weight and mammary tumor latency in MMTV-TGF-alpha mice. Int. J. Obes. Relat. Metab. Disord..

[7] Zhao Y., Tan Y.S., Aupperlee M.D., Langohr I.M., Kirk E.L., Troester M.A., Schwartz R.C., Haslam S.Z. (2013). Pubertal high fat diet: Effects on mammary cancer development. Breast Cancer Res..

[8] Elliott B.E., Tam S.-P., Dexter D., Chen Z.Q. (1992). Capacity of adipose tissue to promote growth and metastasis of a murine mammary carcinoma: Effect of estrogen and progesterone. Int. J. Cancer.

[9] Zhang C., Zhu K., Liu J., Yang M. (2025). The Role of Obesity in the Regulation of Immunosuppressive Cell Infiltration and Immunosurveillance in Cancers. Diseases.

[10] Hildebrandt X., Ibrahim M., Peltzer N. (2023). Cell death and inflammation during obesity: “Know my methods, WAT(son)”. Cell Death Differ..

[11] Protani M., Coory M., Martin J.H. (2010). Effect of obesity on survival of women with breast cancer: Systematic review and meta-analysis. Breast Cancer Res. Treat..

[12] Hunter D.J., Spiegelman D., Adami H.O., Beeson L., van den Brandt P.A., Folsom A.R., Fraser G.E., Goldbohm R.A., Graham S., Howe G.R. (1996). Cohort studies of fat intake and the risk of breast cancer—A pooled analysis. N. Engl. J. Med..

[13] van den Brandt P.A., Spiegelman D., Yaun S.S., Adami H.O., Beeson L., Folsom A.R., Fraser G., Goldbohm R.A., Graham S., Kushi L. (2000). Pooled analysis of prospective cohort studies on height, weight, and breast cancer risk. Am. J. Epidemiol..

[14] Nguyen H.L., Geukens T., Maetens M., Aparicio S., Bassez A., Borg A., Brock J., Broeks A., Caldas C., Cardoso F. (2023). Obesity-associated changes in molecular biology of primary breast cancer. Nat. Commun..

[15] Seo B.R., Bhardwaj P., Choi S., Gonzalez J., Andresen Eguiluz R.C., Wang K., Mohanan S., Morris P.G., Du B., Zhou X.K. (2015). Obesity-dependent changes in interstitial ECM mechanics promote breast tumorigenesis. Sci. Transl. Med..

[16] Hillers-Ziemer L.E., McMahon R.Q., Hietpas M., Paderta G., LeBeau J., McCready J., Arendt L.M. (2020). Obesity Promotes Cooperation of Cancer Stem-Like Cells and Macrophages to Enhance Mammary Tumor Angiogenesis. Cancers.

[17] Kuziel G., Moore B.N., Haugstad G.P., Xiong Y., Williams A.E., Arendt L.M. (2023). Alterations in the mammary gland and tumor microenvironment of formerly obese mice. BMC Cancer.

[18] Ringel A.E., Drijvers J.M., Baker G.J., Catozzi A., Garcia-Canaveras J.C., Gassaway B.M., Miller B.C., Juneja V.R., Nguyen T.H., Joshi S. (2020). Obesity Shapes Metabolism in the Tumor Microenvironment to Suppress Anti-Tumor Immunity. Cell.

[19] Hossain F., Majumder S., David J., Bunnell B.A., Miele L. (2021). Obesity Modulates the Gut Microbiome in Triple-Negative Breast Cancer. Nutrients.

[20] Xia S., Sha H., Yang L., Ji Y., Ostrand-Rosenberg S., Qi L. (2011). Gr-1+ CD11b+ myeloid-derived suppressor cells suppress inflammation and promote insulin sensitivity in obesity. J. Biol. Chem..

[21] Altin J.G., Sloan E.K. (1997). The role of CD45 and CD45-associated molecules in T cell activation. Immunol. Cell Biol..

[22] Cho K.W., Zamarron B.F., Muir L.A., Singer K., Porsche C.E., DelProposto J.B., Geletka L., Meyer K.A., O’Rourke R.W., Lumeng C.N. (2016). Adipose Tissue Dendritic Cells Are Independent Contributors to Obesity-Induced Inflammation and Insulin Resistance. J. Immunol..

[23] McFarland H.I., Nahill S.R., Maciaszek J.W., Welsh R.M. (1992). CD11b (Mac-1): A marker for CD8+ cytotoxic T cell activation and memory in virus infection. J. Immunol..

[24] Tran H.Q., Bretin A., Adeshirlarijaney A., Yeoh B.S., Vijay-Kumar M., Zou J., Denning T.L., Chassaing B., Gewirtz A.T. (2020). “Western Diet”-Induced Adipose Inflammation Requires a Complex Gut Microbiota. Cell Mol. Gastroenterol. Hepatol..

[25] Xu H., Barnes G.T., Yang Q., Tan G., Yang D., Chou C.J., Sole J., Nichols A., Ross J.S., Tartaglia L.A. (2003). Chronic inflammation in fat plays a crucial role in the development of obesity-related insulin resistance. J. Clin. Investig..

[26] Clements V.K., Long T., Long R., Figley C., Smith D.M.C., Ostrand-Rosenberg S. (2018). Frontline Science: High fat diet and leptin promote tumor progression by inducing myeloid-derived suppressor cells. J. Leukoc. Biol..

[27] Pawelec G., Verschoor C.P., Ostrand-Rosenberg S. (2019). Myeloid-Derived Suppressor Cells: Not Only in Tumor Immunity. Front. Immunol..

[28] Movahedi K., Guilliams M., Van den Bossche J., Van den Bergh R., Gysemans C., Beschin A., De Baetselier P., Van Ginderachter J.A. (2008). Identification of discrete tumor-induced myeloid-derived suppressor cell subpopulations with distinct T cell-suppressive activity. Blood.

[29] Damuzzo V., Pinton L., Desantis G., Solito S., Marigo I., Bronte V., Mandruzzato S. (2015). Complexity and challenges in defining myeloid-derived suppressor cells. Cytom. B Clin. Cytom..

[30] Porsche C.E., Delproposto J.B., Geletka L., O’Rourke R., Lumeng C.N. (2021). Obesity results in adipose tissue T cell exhaustion. JCI Insight.

[31] Khan I.M., Dai Perrard X.Y., Perrard J.L., Mansoori A., Wayne Smith C., Wu H., Ballantyne C.M. (2014). Attenuated adipose tissue and skeletal muscle inflammation in obese mice with combined CD4+ and CD8+ T cell deficiency. Atherosclerosis.

[32] McLaughlin T., Liu L.F., Lamendola C., Shen L., Morton J., Rivas H., Winer D., Tolentino L., Choi O., Zhang H. (2014). T-cell profile in adipose tissue is associated with insulin resistance and systemic inflammation in humans. Arter. Thromb. Vasc. Biol..

[33] Morris P.G., Hudis C.A., Giri D., Morrow M., Falcone D.J., Zhou X.K., Du B., Brogi E., Crawford C.B., Kopelovich L. (2011). Inflammation and increased aromatase expression occur in the breast tissue of obese women with breast cancer. Cancer Prev. Res..

[34] Savetsky I.L., Torrisi J.S., Cuzzone D.A., Ghanta S., Albano N.J., Gardenier J.C., Joseph W.J., Mehrara B.J. (2014). Obesity increases inflammation and impairs lymphatic function in a mouse model of lymphedema. Am. J. Physiol. Heart Circ. Physiol..

[35] Nitti M.D., Hespe G.E., Kataru R.P., García Nores G.D., Savetsky I.L., Torrisi J.S., Gardenier J.C., Dannenberg A.J., Mehrara B.J. (2016). Obesity-induced lymphatic dysfunction is reversible with weight loss. J. Physiol..

[36] Kong Y., Yang H., Nie R., Zhang X., Zuo F., Zhang H., Nian X. (2025). Obesity: Pathophysiology and therapeutic interventions. Mol. Biomed..

[37] Rakhra V., Galappaththy S.L., Bulchandani S., Cabandugama P.K. (2020). Obesity and the Western Diet: How We Got Here. Mo. Med..

[38] Kopp W. (2019). How Western Diet And Lifestyle Drive The Pandemic Of Obesity And Civilization Diseases. Diabetes Metab. Syndr. Obes..

[39] Elkins M., Horrelt M., Woods B., Lawton S., Ohsumi T.K., Fleischman A., Taudte V., Chou J. (2025). Overfeeding and overweight rapidly reprogram inflammatory signaling. Clin. Immunol..

[40] Esser N., Legrand-Poels S., Piette J., Scheen A.J., Paquot N. (2014). Inflammation as a link between obesity, metabolic syndrome and type 2 diabetes. Diabetes Res. Clin. Pract..

[41] Womack J., Tien P.C., Feldman J., Shin J.H., Fennie K., Anastos K., Cohen M.H., Bacon M.C., Minkoff H. (2007). Obesity and immune cell counts in women. Metabolism.

[42] Yoshimura A., Ohnishi S., Orito C., Kawahara Y., Takasaki H., Takeda H., Sakamoto N., Hashino S. (2015). Association of Peripheral Total and Differential Leukocyte Counts with Obesity-Related Complications in Young Adults. Obes. Facts.

[43] Ostrand-Rosenberg S. (2018). Myeloid derived-suppressor cells: Their role in cancer and obesity. Curr. Opin. Immunol..

[44] Takahashi K., Nagai N., Ogura K., Tsuneyama K., Saiki I., Irimura T., Hayakawa Y. (2015). Mammary tissue microenvironment determines T cell-dependent breast cancer-associated inflammation. Cancer Sci..

[45] Kiran S., Rakib A., Kodidela S., Kumar S., Singh U.P. (2022). High-fat diet-induced dysregulation of immune cells correlates with macrophage phenotypes and chronic inflammation in adipose tissue. Cells.

[46] Krishnamurty A.T., Turley S.J. (2020). Lymph node stromal cells: Cartographers of the immune system. Nat. Immunol..

[47] Magnuson A.M., Fouts J.K., Regan D.P., Booth A.D., Dow S.W., Foster M.T. (2018). Adipose tissue extrinsic factor: Obesity-induced inflammation and the role of the visceral lymph node. Physiol. Behav..

[48] Magnuson A.M., Regan D.P., Fouts J.K., Booth A.D., Dow S.W., Foster M.T. (2017). Diet-induced obesity causes visceral, but not subcutaneous, lymph node hyperplasia via increases in specific immune cell populations. Cell Prolif..

[49] Lumeng C.N. (2013). Innate immune activation in obesity. Mol. Asp. Med..

[50] King B.C., Blom A.M. (2021). Complement in metabolic disease: Metaflammation and a two-edged sword. Semin. Immunopathol..

[51] Lumeng C.N., Saltiel A.R. (2011). Inflammatory links between obesity and metabolic disease. J. Clin. Investig..

[52] Alic L., Dendinovic K., Papac-Milicevic N. (2024). The complement system in lipid-mediated pathologies. Front. Immunol..

[53] Moreno-Navarrete J.M., Fernández-Real J.M. (2019). The complement system is dysfunctional in metabolic disease: Evidences in plasma and adipose tissue from obese and insulin resistant subjects. Semin. Cell Dev. Biol..

[54] Wu H., Ballantyne C.M. (2017). Skeletal muscle inflammation and insulin resistance in obesity. J. Clin. Investig..

[55] Gao F., Litchfield B., Wu H. (2024). Adipose tissue lymphocytes and obesity. J. Cardiovasc. Aging.

[56] Khan S., Chan Y.T., Revelo X.S., Winer D.A. (2020). The Immune Landscape of Visceral Adipose Tissue During Obesity and Aging. Front. Endocrinol..

[57] Fain J.N. (2010). Release of inflammatory mediators by human adipose tissue is enhanced in obesity and primarily by the nonfat cells: A review. Mediat. Inflamm..

[58] Haka A.S., Barbosa-Lorenzi V.C., Lee H.J., Falcone D.J., Hudis C.A., Dannenberg A.J., Maxfield F.R. (2016). Exocytosis of macrophage lysosomes leads to digestion of apoptotic adipocytes and foam cell formation. J. Lipid Res..

[59] Cinti S., Mitchell G., Barbatelli G., Murano I., Ceresi E., Faloia E., Wang S., Fortier M., Greenberg A.S., Obin M.S. (2005). Adipocyte death defines macrophage localization and function in adipose tissue of obese mice and humans. J. Lipid Res..

[60] Kawai T., Autieri M.V., Scalia R. (2021). Adipose tissue inflammation and metabolic dysfunction in obesity. Am. J. Physiol. Cell Physiol..

[61] Ferrante A.W. (2007). Obesity-induced inflammation: A metabolic dialogue in the language of inflammation. J. Intern. Med..

[62] Wani K., AlHarthi H., Alghamdi A., Sabico S., Al-Daghri N.M. (2021). Role of NLRP3 Inflammasome Activation in Obesity-Mediated Metabolic Disorders. Int. J. Environ. Res. Public Health.

[63] Yin Z., Deng T., Peterson L.E., Yu R., Lin J., Hamilton D.J., Reardon P.R., Sherman V., Winnier G.E., Zhan M. (2014). Transcriptome analysis of human adipocytes implicates the NOD-like receptor pathway in obesity-induced adipose inflammation. Mol. Cell Endocrinol..

[64] Vandanmagsar B., Youm Y.H., Ravussin A., Galgani J.E., Stadler K., Mynatt R.L., Ravussin E., Stephens J.M., Dixit V.D. (2011). The NLRP3 inflammasome instigates obesity-induced inflammation and insulin resistance. Nat. Med..

[65] Nagareddy P.R., Kraakman M., Masters S.L., Stirzaker R.A., Gorman D.J., Grant R.W., Dragoljevic D., Hong E.S., Abdel-Latif A., Smyth S.S. (2014). Adipose tissue macrophages promote myelopoiesis and monocytosis in obesity. Cell Metab..

[66] Karbiener M., Glantschnig C., Pisani D.F., Laurencikiene J., Dahlman I., Herzig S., Amri E.-Z., Scheideler M. (2015). Mesoderm-specific transcript (MEST) is a negative regulator of human adipocyte differentiation. Int. J. Obes..

[67] Nikonova L., Koza R.A., Mendoza T., Chao P.M., Curley J.P., Kozak L.P. (2008). Mesoderm-specific transcript is associated with fat mass expansion in response to a positive energy balance. FASEB J..

[68] Takahashi M., Kamei Y., Ezaki O. (2005). *Mest/Peg1* imprinted gene enlarges adipocytes and is a marker of adipocyte size. Am. J. Physiol. Endocrinol. Metab..

[69] Anunciado-Koza R.P., Manuel J., Mynatt R.L., Zhang J., Kozak L.P., Koza R.A. (2017). Diet-induced adipose tissue expansion is mitigated in mice with a targeted inactivation of mesoderm specific transcript (*Mest*). PLoS ONE.

[70] Voigt A., Ribot J., Sabater A.G., Palou A., Bonet M.L., Klaus S. (2015). Identification of *Mest/Peg1* gene expression as a predictive biomarker of adipose tissue expansion sensitive to dietary anti-obesity interventions. Genes. Nutr..

[71] Ottenhof K.W., Sild M., Lévesque M.L., Ruhé H.G., Booij L. (2018). TPH2 polymorphisms across the spectrum of psychiatric morbidity: A systematic review and meta-analysis. Neurosci. Biobehav. Rev..

[72] van Galen K.A., Ter Horst K.W., Serlie M.J. (2021). Serotonin, food intake, and obesity. Obes. Rev..

[73] Park B.I., Reeves A.R., Zhu Y., Wilson R.A., Fernandes S.C., Buhman K.K., Lytle K.A., Jensen M.D., Greenberg A.S. (2025). Hyperinsulinemia-induced upregulation of adipocyte TPH2 contributes to peripheral serotonin production, metabolic dysfunction, and obesity. J. Clin. Investig..

[74] Yang Y., Cao J., Shi Y. (2004). Identification and Characterization of a Gene Encoding Human *LPGAT1*, an Endoplasmic Reticulum-associated Lysophosphatidylglycerol Acyltransferase*[boxs]. J. Biol. Chem..

[75] Xu Y., Miller P.C., Phoon C.K.L., Ren M., Nargis T., Rajan S., Hussain M.M., Schlame M. (2022). LPGAT1 controls the stearate/palmitate ratio of phosphatidylethanolamine and phosphatidylcholine in sn-1 specific remodeling. J. Biol. Chem..

[76] Sun H., Zhang J., Ye Q., Jiang T., Liu X., Zhang X., Zeng F., Li J., Zheng Y., Han X. (2023). LPGAT1 controls MEGDEL syndrome by coupling phosphatidylglycerol remodeling with mitochondrial transport. Cell Rep..

[77] Traurig M.T., Orczewska J.I., Ortiz D.J., Bian L., Marinelarena A.M., Kobes S., Malhotra A., Hanson R.L., Mason C.C., Knowler W.C. (2013). Evidence for a role of *LPGAT1* in influencing BMI and percent body fat in Native Americans. Obesity.

[78] Su Y.H., Wu Y.Z., Ann D.K., Chen J.L., Kuo C.Y. (2023). Obesity promotes radioresistance through SERPINE1-mediated aggressiveness and DNA repair of triple-negative breast cancer. Cell Death Dis..

[79] Kong H.J., Kwon E.J., Kwon O.S., Lee H., Choi J.Y., Kim Y.J., Kim W., Cha H.J. (2021). Crosstalk between YAP and TGFβ regulates SERPINE1 expression in mesenchymal lung cancer cells. Int. J. Oncol..

[80] Kaji H. (2016). Adipose Tissue-Derived Plasminogen Activator Inhibitor-1 Function and Regulation. Compr. Physiol..

[81] Levine J.A., Oleaga C., Eren M., Amaral A.P., Shang M., Lux E., Khan S.S., Shah S.J., Omura Y., Pamir N. (2021). Role of PAI-1 in hepatic steatosis and dyslipidemia. Sci. Rep..

[82] Zheng Z., Nakamura K., Gershbaum S., Wang X., Thomas S., Bessler M., Schrope B., Krikhely A., Liu R.-M., Ozcan L. (2020). Interacting hepatic PAI-1/tPA gene regulatory pathways influence impaired fibrinolysis severity in obesity. J. Clin. Investig..

[83] Lopez-Legarrea P., Mansego M.L., Zulet M.A., Martinez J.A. (2013). *SERPINE1*, PAI-1 protein coding gene, methylation levels and epigenetic relationships with adiposity changes in obese subjects with metabolic syndrome features under dietary restriction. J. Clin. Biochem. Nutr..

[84] Ma L.-J., Mao S.-L., Taylor K.L., Kanjanabuch T., Guan Y., Zhang Y., Brown N.J., Swift L.L., McGuinness O.P., Wasserman D.H. (2004). Prevention of obesity and insulin resistance in mice lacking plasminogen activator inhibitor 1. Diabetes.

[85] Coffey C.S., Asselbergs F.W., Hebert P.R., Hillege H.L., Li Q., Moore J.H., van Gilst W.H. (2011). The association of the metabolic syndrome with PAI-1 and t-PA levels. Cardiol. Res. Pract..

[86] Mizunoe Y., Kobayashi M., Tagawa R., Nakagawa Y., Shimano H., Higami Y. (2019). Association between Lysosomal Dysfunction and Obesity-Related Pathology: A Key Knowledge to Prevent Metabolic Syndrome. Int. J. Mol. Sci..

[87] Singer K., DelProposto J., Lee Morris D., Zamarron B., Mergian T., Maley N., Cho K.W., Geletka L., Subbaiah P., Muir L. (2014). Diet-induced obesity promotes myelopoiesis in hematopoietic stem cells. Mol. Metab..

[88] Zhang J., Wright W., Bernlohr D.A., Cushman S.W., Chen X. (2007). Alterations of the classic pathway of complement in adipose tissue of obesity and insulin resistance. Am. J. Physiol.-Endocrinol. Metab..

[89] Ruf W., Samad F. (2015). Tissue factor pathways linking obesity and inflammation. Hämostaseologie.

[90] Garin J., Diez R., Kieffer S., Dermine J.-F., Duclos S., Gagnon E., Sadoul R., Rondeau C., Desjardins M. (2001). The phagosome proteome: Insight into phagosome functions. J. Cell Biol..

[91] Ferré P. (2004). The Biology of Peroxisome Proliferator-Activated Receptors: Relationship with Lipid Metabolism and Insulin Sensitivity. Diabetes.

[92] Tyagi S., Gupta P., Saini A.S., Kaushal C., Sharma S. (2011). The peroxisome proliferator-activated receptor: A family of nuclear receptors role in various diseases. J. Adv. Pharm. Technol. Res..

[93] Ahmadian M., Suh J.M., Hah N., Liddle C., Atkins A.R., Downes M., Evans R.M. (2013). PPARγ signaling and metabolism: The good, the bad and the future. Nat. Med..

[94] Sun K., Tordjman J., Clément K., Scherer P.E. (2013). Fibrosis and Adipose Tissue Dysfunction. Cell Metab..

[95] Mariman E.C.M., Wang P. (2010). Adipocyte extracellular matrix composition, dynamics and role in obesity. Cell. Mol. Life Sci..

[96] Hotamisligil G.S. (2006). Inflammation and metabolic disorders. Nature.

[97] Boucher J., Kleinridders A., Kahn C.R. (2014). Insulin receptor signaling in normal and insulin-resistant states. Cold Spring Harb. Perspect. Biol..

[98] Trayhurn P., Wood I.S. (2004). Adipokines: Inflammation and the pleiotropic role of white adipose tissue. Br. J. Nutr..

[99] de Sousa Neto I.V., Durigan J.L.Q., da Silva A.S.R., de Cássia Marqueti R. (2022). Adipose Tissue Extracellular Matrix Remodeling in Response to Dietary Patterns and Exercise: Molecular Landscape, Mechanistic Insights, and Therapeutic Approaches. Biology.

[100] Datta R., Podolsky M.J., Atabai K. (2018). Fat fibrosis: Friend or foe?. JCI Insight.

[101] Marcelin G., Gautier E.L., Clément K. (2022). Adipose Tissue Fibrosis in Obesity: Etiology and Challenges. Annu. Rev. Physiol..

[102] Caruso A., Accattatis F.M., Giordano C., Gelsomino L., Del Console P., Fiorita M.F., Gyorffy B., Bianchi L., Carleo A., De Salvo R. (2025). Adipocyte/Tumor cell crosstalk via IGF-1/TXNIP axis promotes malignancy and endocrine resistance in breast cancer. Cell Commun. Signal..

[103] Accattatis F.M., Caruso A., Carleo A., Del Console P., Gelsomino L., Bonofiglio D., Giordano C., Barone I., Andò S., Bianchi L. (2023). CEBP-β and PLK1 as Potential Mediators of the Breast Cancer/Obesity Crosstalk: In Vitro and In Silico Analyses. Nutrients.

[104] Gelsomino L., Caruso A., Tasan E., Leonetti A.E., Malivindi R., Naimo G.D., Giordano F., Panza S., Gu G., Perrone B. (2024). Evidence that CRISPR-Cas9 Y537S-mutant expressing breast cancer cells activate Yes-associated protein 1 to driving the conversion of normal fibroblasts into cancer-associated fibroblasts. Cell Commun. Signal..

[105] Nieman K.M., Romero I.L., Van Houten B., Lengyel E. (2013). Adipose tissue and adipocytes support tumorigenesis and metastasis. Biochim. Biophys. Acta.

