# Supplementary material for: Western Diet-Induced Obesity Modulates the Mammary Fat Pad Microenvironment
Source: Cells. 2026 Jun 8;15(12):1050. doi: 10.3390/cells15121050 (PMC13297574; doi:10.3390/cells15121050)
Supplement: Supplementary file 1 [file cells-15-01050-s001.zip › Figure_S1.pptx]

## Slide 1
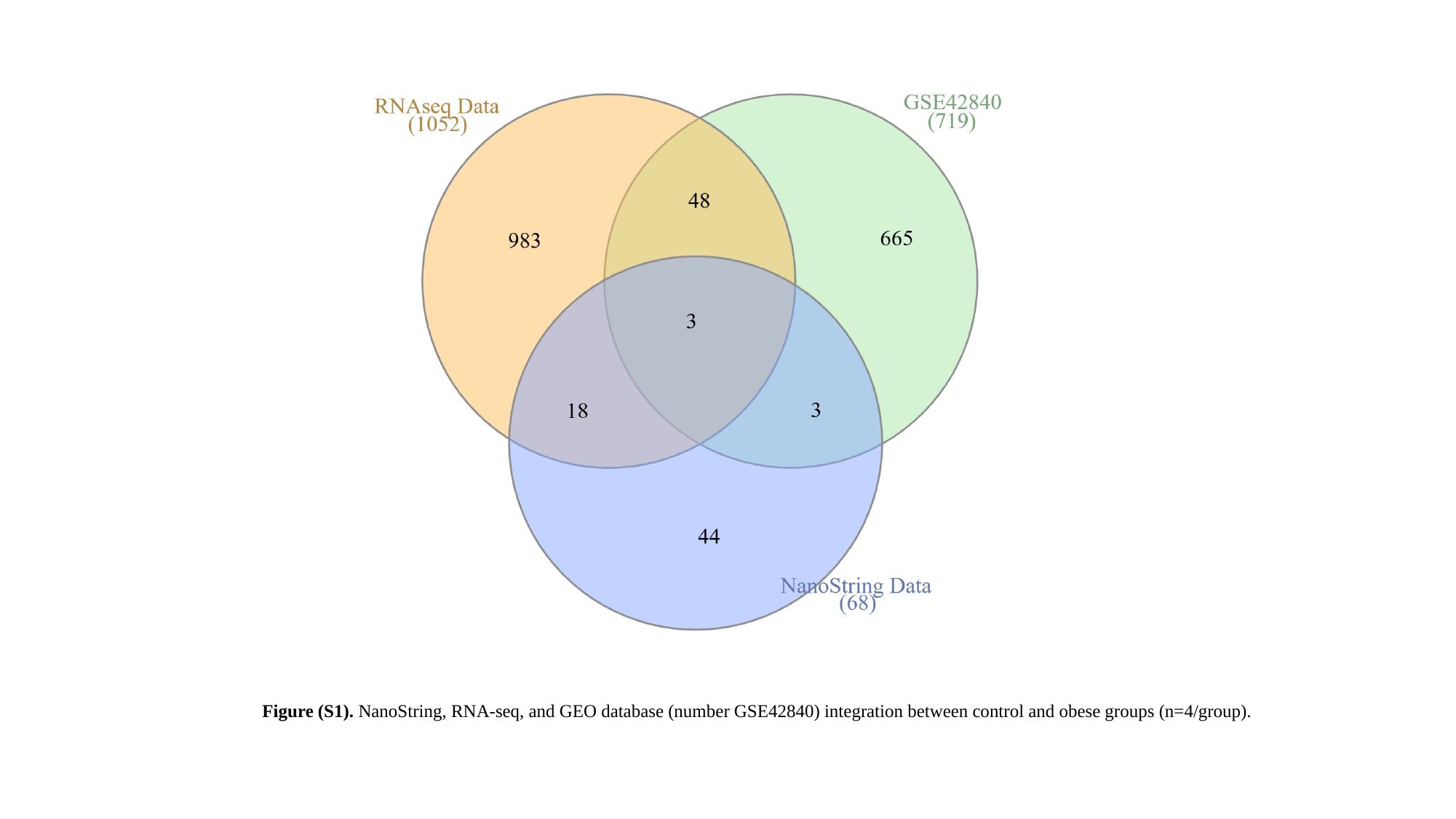

Figure (S1). NanoString, RNA-seq, and GEO database (number GSE42840) integration between control and obese groups (n=4/group).
